# Collective Response of Human Populations to Large-Scale Emergencies

**DOI:** 10.1371/journal.pone.0017680

**Published:** 2011-03-30

**Authors:** James P. Bagrow, Dashun Wang, Albert-László Barabási

**Affiliations:** 1 Center for Complex Network Research, Department of Physics, Northeastern University, Boston, Massachusetts, United States of America; 2 Center for Cancer Systems Biology, Dana-Farber Cancer Institute, Boston, Massachusetts, United States of America; 3 Department of Medicine, Harvard Medical School, Boston, Massachusetts, United States of America; University of Zaragoza, Spain

## Abstract

Despite recent advances in uncovering the quantitative features of stationary human activity patterns, many applications, from pandemic prediction to emergency response, require an understanding of how these patterns change when the population encounters unfamiliar conditions. To explore societal response to external perturbations we identified real-time changes in communication and mobility patterns in the vicinity of eight emergencies, such as bomb attacks and earthquakes, comparing these with eight non-emergencies, like concerts and sporting events. We find that communication spikes accompanying emergencies are both spatially and temporally localized, but information about emergencies spreads globally, resulting in communication avalanches that engage in a significant manner the social network of eyewitnesses. These results offer a quantitative view of behavioral changes in human activity under extreme conditions, with potential long-term impact on emergency detection and response.

## Introduction

Current research on human dynamics is limited to data collected under normal and stationary circumstances [1], capturing the regular daily activity of individuals [2], [3], [4], [5], [6], [7], [8], [9], [10], [11], [12], [13], [14], [15]. Yet, there is exceptional need to understand how people change their behavior when exposed to rapidly changing or unfamiliar conditions [1], such as life-threatening epidemic outbreaks [4], [12], emergencies and traffic anomalies, as models based on stationary events are expected to break down under these circumstances. Such rapid changes in conditions are often caused by natural, technological or societal disasters, from hurricanes to violent conflicts [16]. The possibility to study such real time changes has emerged recently thanks to the widespread use of mobile phones, which track both user mobility [2], [3], [6], [17] and real-time communications along the links of the underlying social network [7], [18]. Here we take advantage of the fact that mobile phones act as *in situ* sensors at the site of an emergency, to study the real-time behavioral patterns of the local population under external perturbations caused by emergencies. Advances in this direction not only help redefine our understanding of information propagation [19] and cooperative human actions under externally induced perturbations, which is the main motivation of our work, but also offer a new perspective on panic [20], [21], [22], [23] and emergency protocols in a data-rich environment [24].

Our starting point is a country-wide mobile communications dataset, culled from the anonymized billing records of approximately ten million mobile phone subscribers of a mobile company which covers about one-fourth of subscribers in a country with close to full mobile penetration. It provides the time and duration of each mobile phone call [7], together with information on the tower that handled the call, thus capturing the real-time locations of the users [3], [6], [25] (Methods, File S1, Fig. A). To identify potential societal perturbations, we scanned media reports pertaining to the coverage area between January 2007 and January 2009 and developed a corpus of times and locations for eight societal, technological, and natural emergencies, ranging from bombings to a plane crash, earthquakes, floods and storms (Table 1). Approximately 30% of the events mentioned in the media occurred in locations with sparse cellular coverage or during times when few users are active (like very early in the morning). The remaining events do offer, however, a sufficiently diverse corpus to explore the generic vs. unique changes in the activity patterns in response to an emergency. Here we discuss four events, chosen for their diversity: (1) a bombing, resulting in several injuries (no fatalities); (2) a plane crash resulting in a significant number of fatalities; (3) an earthquake whose epicenter was outside our observation area but affected the observed population, causing mild damage but no casualties; and (4) a power outage (blackout) affecting a major metropolitan area ( File S1, Fig. B). To distinguish emergencies from other events that cause collective changes in human activity, we also explored eight planned events, such as sports games and a popular local sports race and several rock concerts. We discuss here in detail a cultural festival and a large pop music concert as non-emergency references (Table 1, see also File S1, Sec. B). The characteristics of the events not discussed here due to length limitations are provided in File S1, Sec. I for completeness and comparison.

**Table 1:** Summary of the studied emergencies and non-emergencies.

		Event	duration (hours)	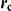 (km)	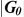	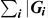
**Emergencies**	1	*Bombing*	1.92	2.38	750	5,099
	2	*Plane crash*	2.17	9.98	2,104	7,325
	3	*Earthquake*	1.42	110	32,403	83,280
	4	*Blackout*	3.0	3.02	84,751	288,332
	5	Jet scare	1.67	6.18	3,556	11,575
	6	Storm 1	2.33	27.0	7,350	18,124
	7	Storm 2	2.0	4.29	14,634	33,963
	8	Storm 3	1.75	2.79	19,239	48,626
**Non-emergencies**	9	*Concert 1*	13.25	0.48	11,376	91,889
	10	Concert 2	6.67	1.06	3,939	29,837
	11	Concert 3	9.08	1.48	5,134	81,125
	12	Concert 4	12.08	0.35	2,630	17,998
	13	*Festival 1*	19.92	0.36	66,869	454,687
	14	Festival 2	2.17	0.50	1,453	7,963
	15	Festival 3	20.92	1.33	10,854	427,839
	16	Festival 4	11.25	0.72	3,117	16,822

**Figure 1:**
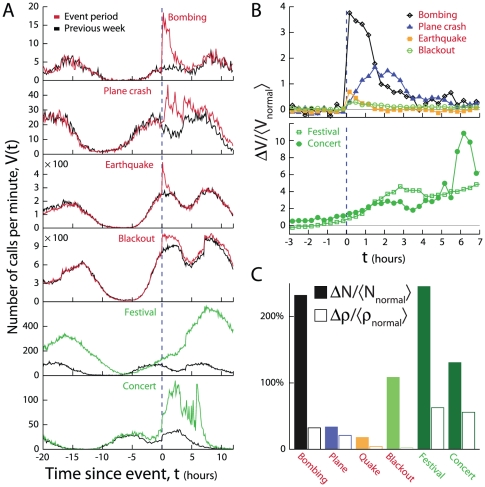
Call anomalies during emergencies. **A**, The time dependence of call volume 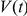 in the vicinity of four emergencies and two non-emergencies (See Table 1). **B**, The temporal behavior of the relative call volume 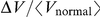 of the events shown in **A**, where 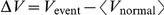, *V*_event_ is the call volume on the day of the event (shown in red in **A**), and 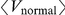 is the average call volume during the same period of the week (the call volume during the previous week is shown in black in **B**). **C**, The relative change in the average number of calls placed per user (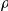) and the total number of users (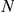) making calls from the region indicates that the call anomaly is primarily due to a significant increase in the number of users that place calls during the events.

**Figure 2 pone-0017680-g002:**
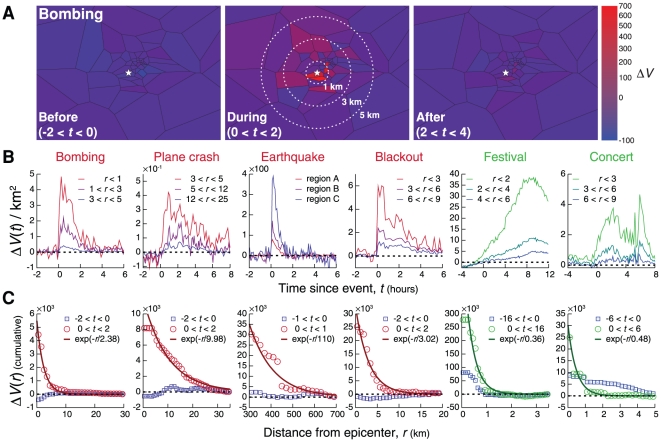
The spatial impact of an emergency. **A**, Maps of total anomalous call activity (activity during the event minus expected normal activity) for two-hour periods before (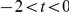), during (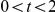), and after (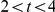) the bombing. The color code corresponds to the total change 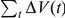, where the sum runs over the particular time period. **B**, Changes in call volume in regions at various distances 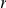 from the event epicenter. Note that the peak of the call volume anomaly for the bombing within the observed 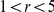 km region is delayed by approximately 10 minutes compared to the 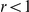 km epicenter region. No call anomaly is observed for 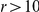 km. The earthquake covers a large spatial range so we instead choose three event regions A–C, at distances of 310 km, 340 km, and 425 km from the seismic epicenter (which was outside the studied region). **C**, To measure the distance dependence of the anomaly, we computed the total anomalous call volume in **B** before (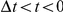) and after (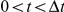) each event as a function of the distance 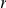, revealing approximately exponential decay, 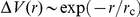. Non-emergencies are spatially localized, with 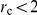 km.

**Figure 3 pone-0017680-g003:**
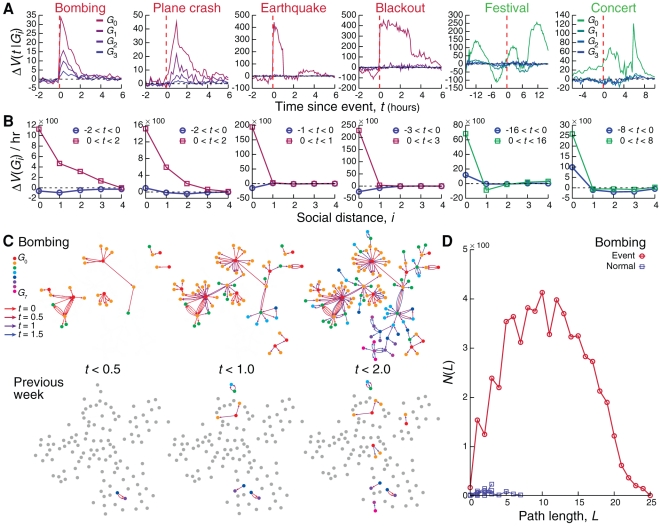
Social characteristics of information cascades. **A**, Changes in call volume for users directly affected by the event (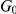), users that receive calls from 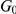 but are not near the event (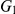), users contacted by 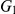 but not in 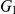 or 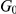 (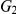), etc. For the bombing and plane crash, populations respond very rapidly, within minutes. **B**, The total amount of anomalous call activity in **A** before (during 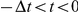) and after (during 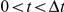) the event for each user group 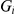 quantifies the impact on the social network. We see that information propagates deeply into the social network for the bombing and plane crash. **C**, Top panel: the contact network formed between affected users during the bombing. Bottom panel: the call pattern between users that are active during the emergency during the previous week, indicating that the information cascade observed during the bombing is out of the ordinary. **D**, The distribution of shortest paths within the contact network quantifies the anomalous information cascade induced by the bombing.

The columns provide the duration of the anomalous call activity (Fig. 1), the spatial decay rate 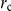 (Fig. 2), the number of users in the event population 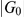, and the total size of the information cascade 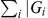 (Fig. 3). Events discussed in the main text are italicized, the rest are discussed in the supplementary material. ‘Jet scare’ refers to a sonic boom interpreted by the local population and initial media reports as an explosion.

## Results and Discussion

As shown in Fig. 1A, emergencies trigger a sharp spike in call activity (number of outgoing calls and text messages) in the physical proximity of the event, confirming that mobile phones act as sensitive local “sociometers” to external societal perturbations. The call volume starts decaying immediately after the emergency, suggesting that the urge to communicate is strongest right at the onset of the event. We see virtually no delay between the onset of the event and the jump in call volume for events that were directly witnessed by the local population, such as the bombing, the earthquake and the blackout. Brief delay is observed only for the plane crash, which took place in an unpopulated area and thus lacked eyewitnesses. In contrast, non-emergency events, like the festival and the concert in Fig. 1A, display a gradual increase in call activity, a noticeably different pattern from the “jump-decay” pattern observed for emergencies. See also File S1, Figs. I and J.

To compare the magnitude and duration of the observed call anomalies, in Fig. 1B we show the temporal evolution of the relative call volume 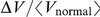 as a function of time, where 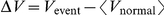, *V*_event_ is the call activity during the event and 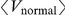 is the average call activity during the same time period of the week. As Fig. 1B indicates, the magnitude of 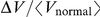 correlates with our relative (and somewhat subjective) sense of the event's potential severity and unexpectedness: the bombing induces the largest change in call activity, followed by the plane crash; whereas the collective reaction to the earthquake and the blackout are somewhat weaker and comparable to each other. While the relative change was also significant for non-emergencies, the emergence of the call anomaly is rather gradual and spans seven or more hours, in contrast with the jump-decay pattern lasting only three to five hours for emergencies (Figs. 1B, File S1, Figs. I and J). As we show in Fig. 1C (see also File S1, Sec. C) the primary source of the observed call anomaly is a sudden increase of calls by individuals who would normally not use their phone during the emergency period, rather than increased call volume by those that are normally active in the area.

The temporally localized spike in call activity (Fig. 1A,B) raises an important question: is information about the events limited to the immediate vicinity of the emergency or do emergencies, often immediately covered by national media, lead to spatially extended changes in call activity [23]? We therefore inspected the change in call activity in the vicinity of the epicenter, finding that for the bombing, for example, the magnitude of the call anomaly is strongest near the event, and drops rapidly with the distance 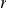 from the epicenter (Fig. 2A). To quantify this effect across all emergencies, we integrated the call volume over time in concentric shells of radius 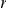 centered on the epicenter (Fig. 2B). The decay is approximately exponential, 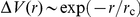, allowing us to characterize the spatial extent of the reaction with a decay rate 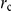 (Fig. 2C). The observed decay rates range from 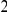 km (bombing) to 10 km (plane crash), indicating that the anomalous call activity is limited to the event's vicinity. An extended spatial range (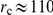 km) is seen only for the earthquake, lacking a narrowly defined epicenter. Meanwhile, a distinguishing pattern of non-emergencies is their highly localized nature: they are characterized by a decay rate of less than 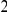 km, implying that the call anomaly was narrowly confined to the venue of the event. This systematic split in 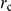 between the spatially extended emergencies and well-localized non-emergencies persists for all explored events (see Table 1, File S1, Fig. K).

Despite the clear temporal and spatial localization of anomalous call activity during emergencies, one expects some degree of information propagation beyond the eyewitness population [26]. We therefore identified the individuals located within the event region 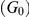, as well as a 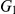 group consisting of individuals outside the event region but who receive calls from the 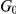 group during the event, a 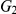 group that receive calls from 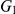, and so on. We see that the 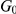 individuals engage their social network within minutes, and that the 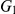, 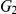, and occasionally even the 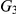 group show an anomalous call pattern immediately after the anomaly (Fig. 3A). This effect is quantified in Fig. 3B, where we show the increase in call volume for each group as a function of their social network based distance from the epicenter (for example, the social distance of the 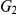 group is 2, being two links away from the 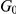 group), indicating that the bombing and plane crash show strong, immediate social propagation up to the third and second neighbors of the eyewitness 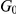 population, respectively. The earthquake and blackout, less threatening emergencies, show little propagation beyond the immediate social links of 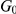 and social propagation is virtually absent in non-emergencies.

The nature of the information cascade behind the results shown in Fig. 3A,B is illustrated in Fig. 3C, where we show the individual calls between users active during the bombing. In contrast with the information cascade triggered by the emergencies witnessed by the 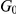 users, there are practically no calls between the same individuals during the previous week. To quantify the magnitude of the information cascade we measured the length of the paths emanating from the 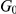 users, finding them to be considerably longer during the emergency (Fig. 3D), compared to five non-emergency periods, demonstrating that the information cascade penetrates deep into the social network, a pattern that is absent during normal activity [27]. See also File S1, Figs. E, F, G, H, L, M, N, and O, and Table A.

The existence of such prominent information cascades raises tantalizing questions about who contributes to information propagation about the emergency. Using self-reported gender information available for most users (see File S1), we find that during emergencies female users are more likely to make a call than expected based on their normal call patterns. This gender discrepancy holds for the 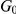 (eyewitness) and 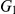 groups, but is absent for non-emergency events (see File S1, Sec. E, Fig. C). We also separated the total call activity of 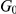 and 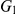 individuals into voice and text messages (including SMS and MMS). For most events (the earthquake and blackout being the only exceptions), the voice/text ratios follow the normal patterns ( File S1, Fig. D), indicating that users continue to rely on their preferred means of communication during an emergency.

**Figure 4:**
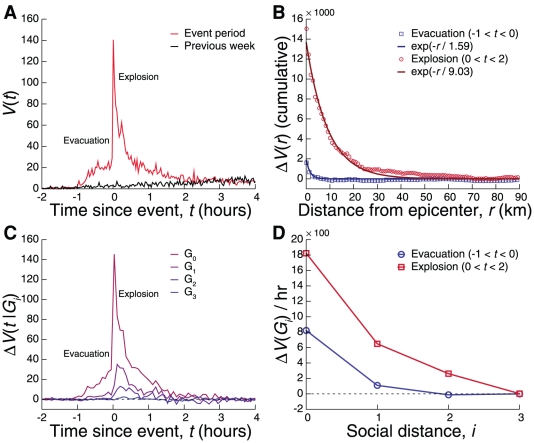
Analyzing a composite event (evacuation preceding an explosion). **A**, Call activity increases during the evacuation (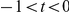) but levels off after the initial warning, until the explosion at 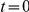 causes a much larger increase in call activity. **B**, Spatially, the evacuation causes a sharply localized activity spike (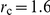 km), but the explosion increases the spatial extent dramatically (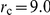 km). **C–D**, The evacuation only activates the 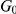 (eyewitness) and 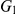 groups, meaning that information fails to propagate significantly beyond the initial group and their immediate ties. However, the blast not only leads to a further increase in call activity in the 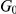 and 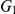 groups, but also triggers the second neighbors 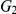.

The patterns identified discussed above allow us to dissect complex events, such as an explosion in an urban area preceded by an evacuation starting approximately one hour before the blast. While a call volume anomaly emerges right at the start of the evacuation, it levels off and the jump-decay pattern characteristic of an emergency does not appear until the real explosion (Fig. 4A). The spatial extent of the evacuation response is significantly smaller than the one observed during the event (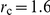 for the evacuation compared with 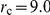 for the explosion, see Fig. 4B). During the evacuation, social propagation is limited to the 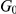 and 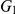 groups only (Fig. 4C,D) while after the explosion we observe a communication cascade that activates the 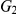 users as well. The lack of strong propagation during evacuation indicates that individuals tend to be reactive rather than proactive and that a real emergency is necessary to initiate a communication cascade that effectively spreads emergency information.

The results of Figs. 1–4 not only indicate that the collective response of the population to an emergency follows reproducible patterns common across diverse events, but they also document subtle differences between emergencies and non-emergencies. We therefore identified four variables that take different characteristic values for emergencies and non-emergencies: (i) the midpoint fraction 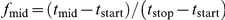, where 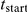 and 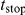 are the times when the anomalous activity begins and ends, respectively, and 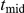 is the time when half of the total anomalous call volume has occurred; (ii) the spatial decay rate 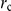 capturing the extent of the event; (iii) the relative size 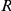 of each information cascade, representing the ratio between the number of users in the event cascade and the cascade tracked during normal periods; (iv) the probability for users to contact existing friends (instead of placing calls to strangers).

**Figure 5:**
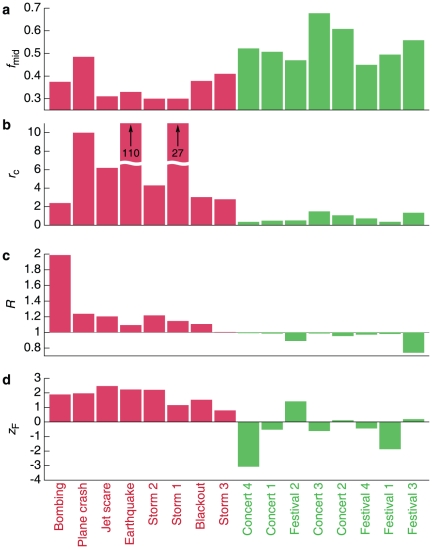
Systematic response mechanisms during emergencies. **A**, The midpoint fraction 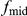 quantifying the onset speed of anomalous call activity (a lower 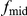 indicates a faster onset). Emergencies display a more abrupt call anomaly than non-emergencies, which feature gradual buildups of anomalous call activity. **B**, The spatial extent of the events, quantified by 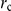, indicates that non-emergency events are far more centrally localized than unexpected emergencies. **C**, The relative cascade size 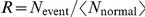, where 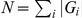 is the number of users in the social cascade. **D**, 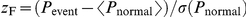, where 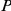 is the probability of calling an acquaintance and 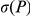 is the standard deviation of 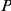.

In Fig. 5 we show these variables for all 16 events, finding systematic differences between emergencies and non-emergencies. As the figure indicates, a multidimensional variable, relying on the documented changes in human activity, can be used to automatically distinguish emergency situations from non-emergency induced anomalies. Such a variable could also help real-time monitoring of emergencies [24], from information about the size of the affected population, to the timeline of the events, and could help identify mobile phone users capable of offering immediate, actionable information, potentially aiding search and rescue.

Rapidly-evolving events such as those studied throughout this work require dynamical data with ultra-high temporal and spatial resolution and high coverage. Although the populations affected by emergencies are quite large, occasionally reaching thousands of users, due to the demonstrated localized nature of the anomaly, this size is still small in comparison to other proxy studies of human dynamics, which can exploit the activity patterns of millions of internet users or webpages [13], [14], [15], [27]. Meanwhile, emergencies occur over very short timespans, a few hours at most, whereas much current work on human dynamics relies on longitudinal datasets covering months or even years of activity for the same users (e.g. [3], [6], [9]), integrating out transient events and noise. But in the case of emergencies, such transient events are precisely what we wish to quantify. Given the short duration and spatially localized nature of these events, it is vital to have extremely high coverage of the entire system, to maximize the availability of critical information during an event. To push human dynamics research into such fast-moving events requires new tools and datasets capable of extracting signals from limited data. We believe that our research offers a first step in this direction.

In summary, similar to how biologists use drugs to perturb the state of a cell to better understand the collective behavior of living systems, we used emergencies as external societal perturbations, helping us uncover generic changes in the spatial, temporal and social activity patterns of the human population. Starting from a large-scale, country-wide mobile phone dataset, we used news reports to gather a corpus of sixteen major events, eight unplanned emergencies and eight scheduled activities. Studying the call activity patterns of users in the vicinity of these events, we found that unusual activity rapidly spikes for emergencies in contrast with non-emergencies induced anomalies that build up gradually before the event; that the call patterns during emergencies are exponentially localized regardless of event details; and that affected users will only invoke the social network to propagate information under the most extreme circumstances. When this social propagation does occur, however, it takes place in a very rapid and efficient manner, so that users three or even four degrees from eyewitnesses can learn of the emergency within minutes.

These results not only deepen our fundamental understanding of human dynamics, but could also improve emergency response. Indeed, while aid organizations increasingly use the distributed, real-time communication tools of the 21st century, much disaster research continues to rely on low-throughput, post-event data, such as questionnaires, eyewitness reports [28], [29], and communication records between first responders or relief organizations [30]. The emergency situations explored here indicate that, thanks to the pervasive use of mobile phones, collective changes in human activity patterns can be captured in an objective manner, even at surprisingly short time-scales, opening a new window on this neglected chapter of human dynamics.

## Materials and Methods

### Dataset

We use a set of anonymized billing records from a western european mobile phone service provider [7], [3], [6]. The records cover approximately 10M subscribers within a single country over 3 years of activity. Each billing record, for voice and text services, contains the unique identifiers of the caller placing the call and the callee receiving the call; an identifier for the cellular antenna (tower) that handled the call; and the date and time when the call was placed. Coupled with a dataset describing the locations (latitude and longitude) of cellular towers, we have the approximate location of the caller when placing the call. For full details, see File S1, Sec. A.

### Identifying events

To find an event in the mobile phone data, we need to determine its time and location. We have used online news aggregators, particularly the local news.google.com service to search for news stories covering the country and time frame of the dataset. Keywords such as ‘storm’, ‘emergency’, ‘concert’, etc. were used to find potential news stories. Important events such as bombings and earthquakes are prominently covered in the media and are easy to find. Study of these reports, which often included photographs of the affected area, typically yields precise times and locations for the events. Reports would occasionally conflict about specific details, but this was rare. We take the *reported* start time of the event as 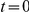.

To identify the beginning and ending of an event, 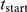 and 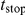, we adopt the following procedure. First, identify the event region (a rough estimate is sufficient) and scan all its calls during a large time period covering the event (e.g., a full day), giving 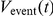. Then, scan calls for a number of “normal” periods, those modulo one week from the event period, exploiting the weekly periodicity of 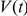. These normal periods' time series are averaged to give 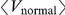. (To smooth time series, we typically bin them into 5–10 minute intervals.) The standard deviation 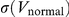 as a function of time is then used to compute 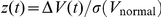. Finally, we define the interval 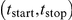 as the longest contiguous run of time intervals where 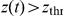, for some fixed cutoff 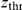. We chose 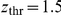 for all events.

For full details, see File S1, Sec. B.

## Supporting Information

File S1 **Supplementary text and figures.**
(PDF)
