# Supplementary material for: Collective Response of Human Populations to Large-Scale Emergencies
Source: PLoS One. 2011 Mar 30;6(3):e17680. doi: 10.1371/journal.pone.0017680 (PMC3068136; doi:10.1371/journal.pone.0017680)
Supplement: File S1 — Supplementary text and figures. (PDF) [file pone.0017680.s001.pdf]

Supporting Information

Collective response of human populations to large-scale emergencies
by James P. Bagrow, Dashun Wang, and Albert-László Barabási

Table of Contents

A Dataset **S2**
A.1 Market share S2

B Identifying events **S2**
B.1 Missing events S3

C Source of call anomaly **S4**

D Whom do people call **S4**

E Gender response during events **S5**

F Voice versus text usage during events **S5**

G Systematic response mechanisms during emergencies **S6**

H Calculating social propagation **S7**
H.1 Constructing the time-dependent contact network S7
H.2 Controlling for social propagation S8

I Results on the event corpus **S11**

List of Figures

A Regional and national visibility of events S3
B Spatial changes in call activity due to the blackout S3
C Gender response during emergency and non-emergency events S5
D Voice and text usage during emergencies S6
E Extracting the time-dependent contact network from call data S7
F Distribution of shortest paths within bombing's contact network S8
G Understanding selection bias S9
H Controlling factors that affect the relative cascade size S11
I Regional call activity for the eight emergencies S12
J Regional call activity for the eight controls S13
K Spatial call activity for remaining events S13
L Social propagation for emergencies 1–4 . . . . . S14
M Social propagation for emergencies 5–8 . . . . . S14
N Social propagation for concerts S15
O Social propagation for festivals S15

List of Tables

A Global Activity Level and stop times for all sixteen events S11

A Dataset

We use a set of anonymized billing records from a western european mobile phone service provider [1, 2, 3]. The records cover approximately 10M subscribers within a single country over 3 years of activity. Each billing record, for voice and text services, contains the unique identifiers of the caller placing the call and the callee receiving the call; an identifier for the cellular antenna (tower) that handled the call; and the date and time when the call was placed. Coupled with a dataset describing the locations (latitude and longitude) of cellular towers, we have the approximate location of the caller when placing the call. Unless otherwise noted, a "call" can be either voice or text (SMS, MMS, etc.), and "call volume" or "call activity" is both voice calls and text messages.

After identifying the start time and location of an event, we then scan these billing records to determine the activity of nearby users. The mobile phone activity patterns of the affected users can then be followed in the weeks preceding or following the event, to provide control or baseline behavior.

Self-reported gender information is available for approximately 90% of subscribers.

A.1 Market share

These records cover approximately 20% of the country's mobile phone market. However, we also possess identification numbers for phones that are outside the provider but that make or receive calls to users within the company. While we do not possess any other information about these lines, nor anything about their users or calls that are made to other numbers outside the service provider, we do have records pertaining to all calls placed to or from these ID numbers involving subscribers covered by our dataset. This information was used to study social propagation (see Sec. H.1).

B Identifying events

To find an event in the mobile phone data, we need to determine its time and location. We have used online news aggregators, particularly the local `news.google.com` service to search for news stories covering the country and time frame of the dataset. Keywords such as 'storm', 'emergency', 'concert', etc. were used to find potential news stories. Important events such as bombings and earthquakes are prominently covered in the media and are easy to find. Study of these reports, which often included photographs of the affected area, typically yields precise times and locations for the events. Reports would occasionally conflict about specific details, but this was rare. We take the *reported* start time of the event as $t = 0$.

Most events are spatially localized, so it is important to consider only the immediate event region. Otherwise, the event signal is masked by normal activity (Fig. A). The local event region can be estimated as a circle centered on the identified epicenter with a radius chosen based on r_c. For the blackout, however, we chose all towers within the affected city (contained within the city's postal codes).

To identify the beginning and the end of an event, t_{start} and t_{stop}, we adopt the following procedure. First, identify the event region (a rough estimate is sufficient) and scan all its calls during a large time period covering the event (e.g., a full day), giving $V_{\text{event}}(t)$. Then, scan calls for a number of "normal" periods, those modulo one week from the event period, exploiting the weekly periodicity of $V(t)$. These

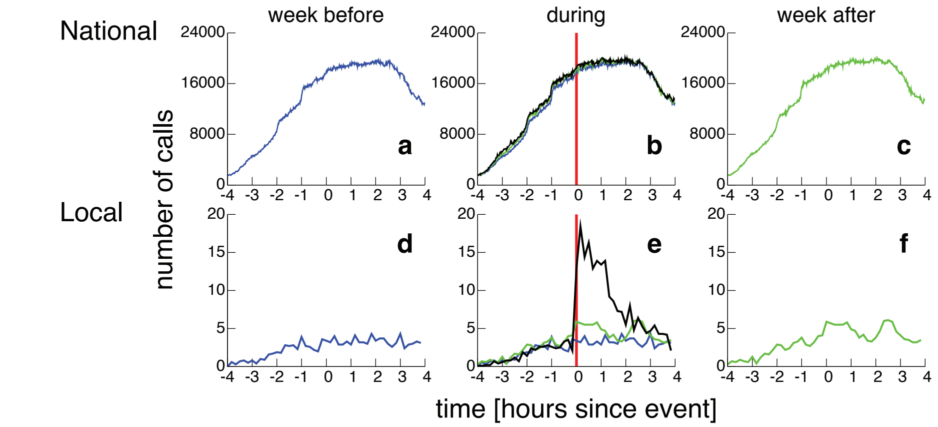

Figure A: **Regional and national visibility of events.** On a national level (**a–c**), the spike in call activity due to the bombing is lost, but it clearly emerges when we focus only on the immediately local vicinity of the event (**d–f**). The strong weekly periodicity in $V(t)$ is also visible.

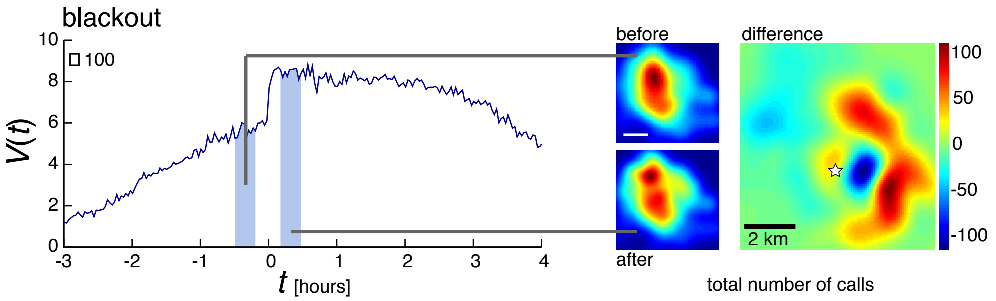

Figure B: **Spatial changes in call activity due to the blackout.** We integrate call activity $V(t)$ over two time windows before and after the blackout occurs (**left**, shaded). Studying total call load spatially we see a nonlinear response, with a region near the city center (star) suffering a drop in calls due to the blackout (**right**). This region is surrounded by areas that display a significant increase in calls, implying that most load was shifted onto nearby cell towers and was not lost.

normal periods' time series are averaged to give $\langle V_{\mathrm{normal}}\rangle$. (To smooth time series, we typically bin them into 5–10 minute intervals.) The standard deviation $\sigma(V_{\mathrm{normal}})$ as a function of time is then used to compute $z(t) = \Delta V(t)/\sigma(V_{\mathrm{normal}})$. Finally, we define the interval $\left(t_{\mathrm{start}}, t_{\mathrm{stop}}\right)$ as the longest contiguous run of time intervals where $z(t) > z_{\mathrm{thr}}$, for some fixed cutoff z_{thr}. We chose $z_{\mathrm{thr}} = 1.5$ for all events.

Finally, there is also the concern that an emergency may be so severe that it interferes with the operations of the mobile phone system itself. Only the blackout caused any damage to the mobile phone system, where some towers were temporarily disabled. (No calls appear to have been lost as other towers picked up the slack.) See Fig. B. Likewise, no towers reached maximum capacity, preventing important calls from being routed. Such effects may occur during larger, more serious emergencies.

B.1 Missing events

It is possibile that newsworthy events may not be discoverable using mobile phones. Indeed, while there are sixteen events documented in main text Table 1, there were a number of events discovered in news reports

that we could not identify in the data. The majority of these were forest fires. While they affected large regions and numbers of people, we could not find them with mobile phones. A large wind storm and a gas main explosion were also not confirmed; both occurred late at night. Two other events, a chemical leak causing an evacuation and a fire at a remote factory causing noxious fumes were discovered in the dataset, but the affected populations were very small, so we decided to discount them.

The absence of these events in the dataset provides important information about the strengths and weaknesses of using mobile phones to study emergencies. Since they rely on user activity, events that occur late at night, when most people are asleep, may be difficult to study. Likewise, events that are severe but diffuse, providing a slight effect over a very broad area, may not be distinguishable from the background of normal activity (although the earthquake is an exception to this). Events in remote locations with little cellular coverage will also be more difficult to study than events in well-covered and well-populated regions.

Finally, since we are especially interested in studying how information propagates socially, we avoided national events, such as televised sports matches or popular public holidays, as these make distinguishing the different event populations G_i unreliable.

C Source of call anomaly

The anomalous call activity raises an important question: is the observed spike due to individuals who normally do not use their phone in the event region and now suddenly choose to place calls, or are those who normally use their phone in the respective timeframe prompted to call more frequently than under normal circumstances? We determined in the event region (i) the relative change in the average number of calls placed per user, $\Delta\rho/\langle\rho_{\mathrm{normal}}\rangle$, and (ii) the relative increase in the number of individuals that use their phone in this period $\Delta N/\langle N_{\mathrm{normal}}\rangle$. Figure 1C shows that during the bombing we see a 36% increase in phone usage whereas the number of individuals that make a call increases by 232%. Other emergencies show a similar pattern: the plane crash, earthquake and blackout show increases in ρ (N) of 21% (67.5%), 1.36% (17.4%) and 4.97% (20.8%), respectively. Taken together, these results indicate that the primary source of the observed call anomaly is a sudden increase of calls by individuals who would normally not use their phone during the emergency period, a behavioral change triggered by the witnessed event.

D Whom do people call

To see if affected users tend to call existing friends or contact strangers, we measured the probability P for a user in G_0 to make his first call between t_{start} and t_{stop} to a friend, where 'friends' are the set of individuals that have had phone contact with the G_0 user during the previous full three months (not including the month of the event). Computing the mean and standard deviation of P over normal time periods (all weekdays of the month of the event, except the day of the event) allows us to quantify the relative change during the event with

$$z_{\mathrm{F}} = \frac{P_{\mathrm{event}} - \langle P_{\mathrm{normal}}\rangle}{\sigma(P_{\mathrm{normal}})}. \tag{S1}$$

In all emergencies we observe an increase in the number of calls placed to friends (and a corresponding decrease in calls to non-friends). Many non-emergencies show the opposite trend: users are less likely to call a friend, although this change is seldom large. See Sec. G and Fig. 5d.

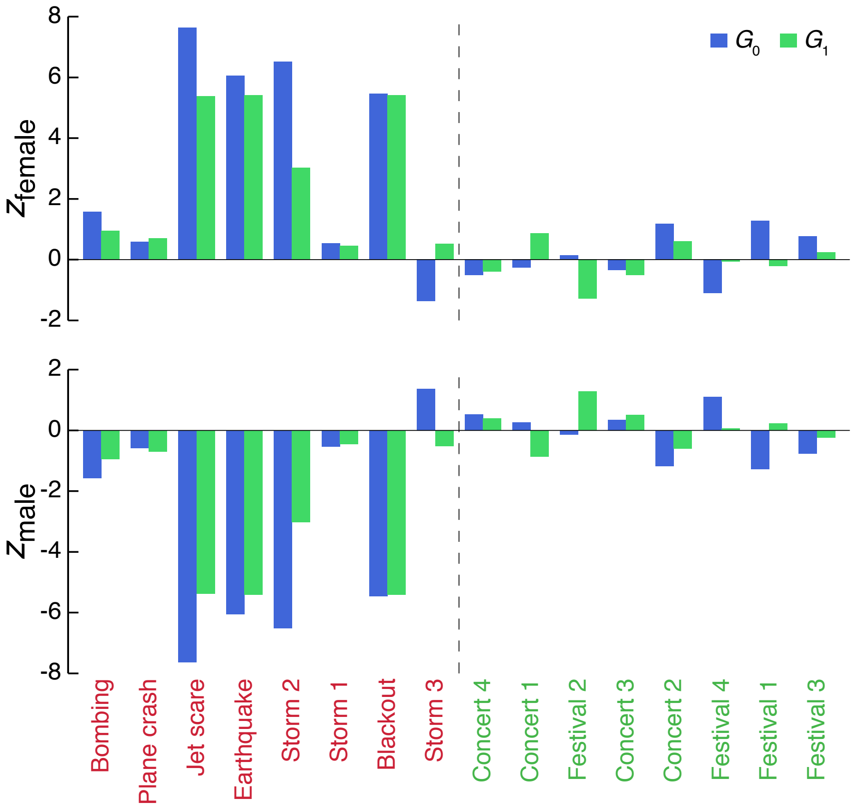

Figure C: **Gender response during emergency and non-emergency events.** For each event we compute the significance z_{female} and z_{male} in the fraction of female and male users active during the event, compared with normal time periods, for both directly affected users (G_0) and those one step away (G_1). We see that nearly all emergencies cause an increase in the fraction of affected female users; this increase is significant for half of the emergencies. Non-emergencies do not result in deviations in the gender breakdown of affected populations.

E Gender response during events

To investigate how the population response to events depends on demographic factors, we used the self-reported gender information, available for the majority ($\sim$88%) of the users. For each event we compute the significance z_{female} and z_{male} in the fraction of female and male users active during the event, compared with normal time periods, as described in D, for both directly affected users (G_0) and those one step away (G_1). As shown in Fig. C, we see that nearly all emergencies cause an increase in the fraction of affected female users; this increase is significant for half of the emergencies. Non-emergencies do not result in deviations in the gender breakdown of affected populations. These results hold for both the directly affected users and their neighbors one step away.

F Voice versus text usage during events

Similar to the quantities z_{F} and z_{female}, we can assess whether users have changed their means of communication due to an event. To do so we compute the significance z_{voice} of the fraction of voice calls compared

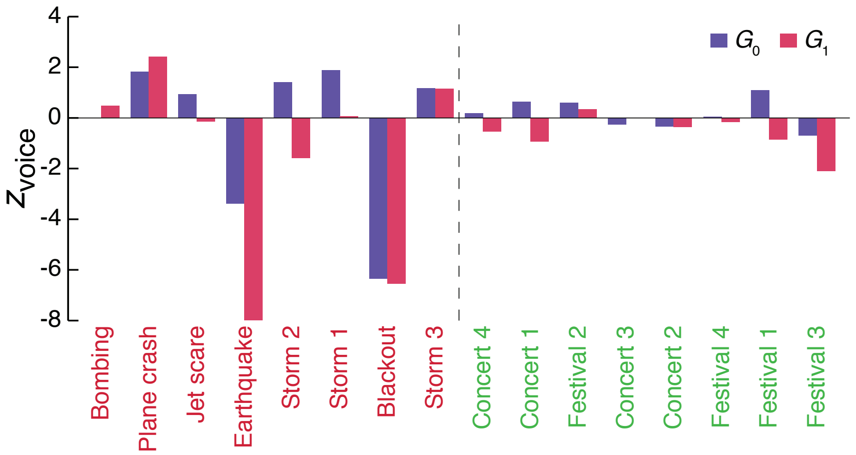

Figure D: **Voice and text usages during emergencies.** Most events do not show a significant change in the fraction of voice calls compared to text messages. The earthquake and blackout are exceptions, as is the plane crash and festival 3 (G_1 only).

to text messages during the event, for both populations G_0 and G_1 (Fig. D). The earthquake and blackout give significantly increased text usage while the plane crash shows an increase in voice, while most other events do not show a significant change. Since the earthquake and blackout were both relatively minor (low danger) events, this result implies that a spike in primarily text messaging activity may indicate that the event is a low-threat/non-critical emergency.

G Systematic response mechanisms during emergencies

As mentioned in the main text, to summarize our current understanding of these events, we computed temporal, spatial, and social properties for each anomaly, plotted in main text Fig. 5. Temporally, we study the midpoint fraction:

$$f_{\mathrm{mid}} = \left(t_{\mathrm{mid}} - t_{\mathrm{start}}\right) / \left(t_{\mathrm{stop}} - t_{\mathrm{start}}\right), \tag{S2}$$

the fraction of time required for half of the anomalous call activity to occur, where t_{mid} satisfies:

$$\int_{t_{\mathrm{start}}}^{t_{\mathrm{mid}}} \left(V_{\mathrm{event}}(t) - V_{\mathrm{normal}}(t)\right) dt = \frac{1}{2} \int_{t_{\mathrm{start}}}^{t_{\mathrm{stop}}} \left(V_{\mathrm{event}}(t) - V_{\mathrm{normal}}(t)\right) dt. \tag{S3}$$

The midpoint fraction is more robust to noisy and non-sharply peaked time series, where estimating t_{peak} is difficult, than the peak fraction. Spatially, we use the anomaly's r_c. Socially, we compute two quantities: the relative size of the cascade R (the number of people in the event cascade divided by the number generated under normal periods; see Sec. H.2 and Fig. H) and z_F, the significance in the probability of calling a friend compared with a non-friend (see Sec. D).

Figure 5 shows a distinct separation in these measures between emergencies and non-emergencies, indicating that there are universal response patterns underlying societal dynamics independent of the particular event details.

a

time	caller	callee	tower	type
2	*i*	*k*	T1	V
3	*j*	*l*	T2	T
5	*k*	*i*	T10	V
7	*l*	*m*	T12	V
8	*l*	*n*	T12	V
9	*l*	*o*	T12	T
12	*m*	*l*	T21	V
14	*n*	*p*	T45	V

event

b

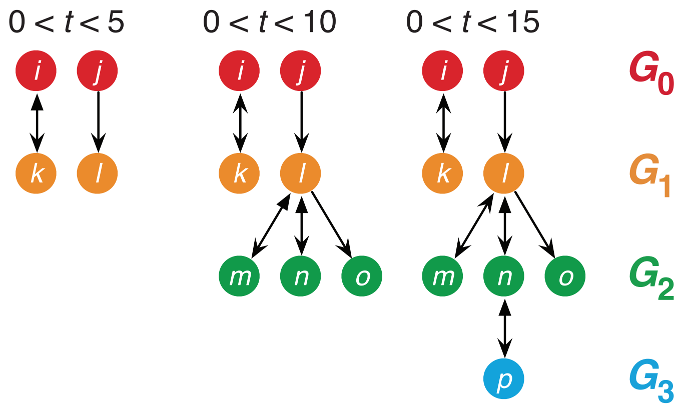

Figure E: **Extracting the time-dependent contact network from call data**. **A,** A cartoon example of the dataset's call records, representing eight calls during 15 minutes following an event. The region of the event contains two towers, T1 and T2. Call type is (T) for text message and (V) for voice call. **B,** Three instances of the evolving contact network, extracted from the example call log shown in **a**. Two users made calls from the region during the first five timesteps, initiating a cascade.

H Calculating social propagation

In this section we detail the procedure for extracting the contact network between users after an event (Sec. H.1) and how to control for various factors to demonstrate whether or not the contact network or its information cascade is anomalous due to the event (Sec. H.2).

H.1 Constructing the time-dependent contact network

We use the following process to generate the contact network between users due to an event. We follow all messages in order of occurrence during the event's time interval $\left[t_{\text{start}}, t_{\text{stop}}\right]$. While we do not know the content of the messages, we assume that any related messages do transmit information pertaining to the event. A user u who does not know about the event becomes "infected" with knowledge due to communication at some time $t \in \left[t_{\text{start}}, t_{\text{stop}}\right]$ if (1) u initiated communication from a tower within the event region or (2) u communicated with a user that was already infected. We place u in the set G_0 if u communicated from the event region, otherwise we place u into G_{i+1} where the infected user transmitting knowledge to u was in G_i. Following these calls and generating the G_i with this procedure then forms the contact network. Note that there are two types of communications in the dataset, voice and text. We assume that voice is bidirectional whereas text messages are not (a user who sends a text message to someone with knowledge of the event will learn nothing from that particular communication). An illustration of this process is depicted in Fig. E.

The contact network itself can be studied using a number of network science tools. One way to analyze the size and scale of this network is through the distribution of shortest (or geodesic) path lengths [4]. In Fig. 3D we presented the distribution of paths emanating from G_0 users within the giant connected component (GCC) of the bombing's network. One can also analyze the distribution for all users, not just G_0, and for all components of the network. These possibilities are shown in Fig. F.

Finally, the cascade of information through a contact network is a non-local process which may be highly effected by sampling/percolation [4]. Indeed, the mobile phone dataset contains only users of a single phone company, and a number of propagation paths may be missing. However, the dataset actually contains all

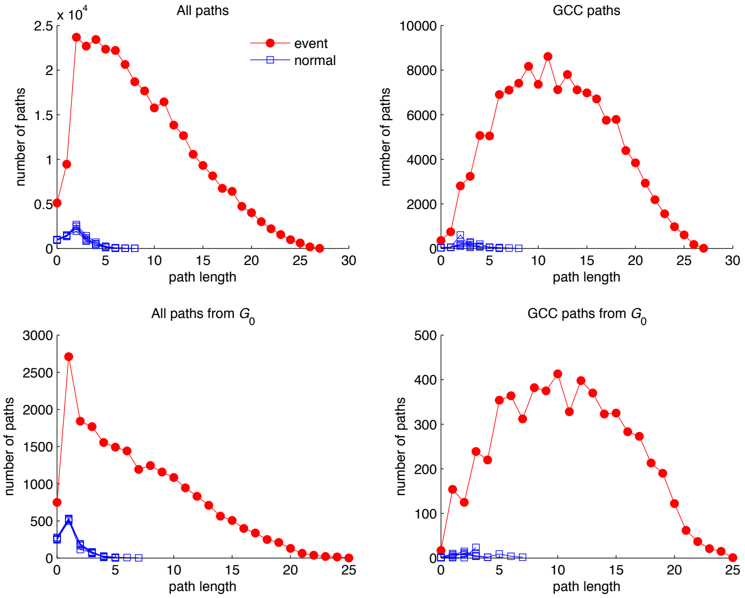

Figure F: **Distributions of shortest paths found within the bombing's contact network.** One can compute shortest paths emanating from all users (**top**) or only users in G_0 (**bottom**) and for paths within the entire network (**left**) or only the network's giant component (**right**).

users who make or receive calls to users within the company, even those outside the company. This means we have all cascade paths of one or two steps that begin and end with in-company users (regardless of whether they travel through a company user or not) and that those paths are the actual shortest paths, providing an effective lower bound on the cascade. In other words, if we can demonstrate the existence of a cascade over users $\{G_0, G_1, G_2\}$, then the actual cascade containing those users can only be larger.

H.2 Controlling for social propagation

A contact network between users can always be constructed, even when no event takes place. There may appear to be propagating cascades as well, since there are temporal correlations between users receiving and then placing calls. This must be properly controlled for.

Suppose we are studying an event and have identified its affected users G_0, those users that made calls during some time window $(t, t+\Delta t)$. We expect that G_0 users will call other users (G_1) outside the event, G_1 users will call G_2 users, etc., generating a cascade $\{G_0, G_1, \ldots\}$. Tracking calls starting from some G will always generate such groups, even during normal periods. Then the question is, do the users in $G_i, i > 0$ show increased activity during an emergency or other event? If so, that is evidence of the social propagation of situational awareness.

To answer this question we need to consider several points:

- Suppose that the total call activity for a region is $V(t)$ (Fig. GA). Now select a group of users $G_{\Delta t}$ that each place at least one call during a small time window Δt. Tracking only their call activity generates the conditional time series $V(t|G_{\Delta t})$. This time series has a "selection bias" that creates the appearance of a large increase in activity during the time window since all the users must place calls then (Fig. GB). This must be accounted for when studying selected users during an event.

- When tracking $V(t|G)$ for a group of $N = |G|$ users, the overall level of activity will depend on N (Fig. GC). A rescaling is necessary when comparing the activity levels of different size groups.

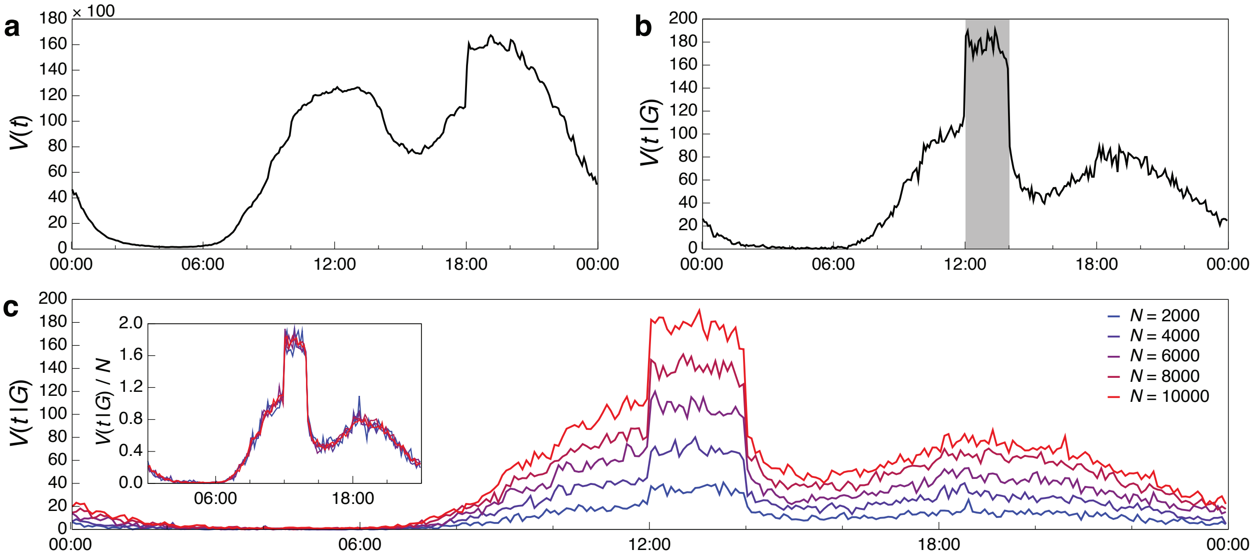

Figure G: **Understanding selection bias. A,** The call volume $V(t)$ of a major city during an ordinary 24-hour period. **B,** The call volume $V(t|G)$ of $N = 10^4$ randomly selected users from **A** who all placed one or more calls between 12:00 and 14:00 (highlighted). The 'bias' of this conditional time series is clear. **C,** The same as **B** for different values of N. Rescaling by the population size (inset) indicates that the relative scale of the bias of $V(t|G)$ during the time window is independent of the population size.

- The selection bias will also depend on the length of the time window Δt. If $\Delta t = 24$ hours had been used in Fig. G, no bias would be evident. All events are compared to time periods with the same Δt, so this effect is automatically controlled for.

Having considered these aspects, we turn our attention to the problem of calculating the cascade itself. For the event period, tracking the outgoing calls of $\{G_0, G_1, \ldots\}$ is straightforward. To determine how unusual this activity is, we need controls for comparison. There are several possibilities:

Control 1 One option is scanning the event region during the same time of the week, collecting a control population of users, and then following their cascade. However, this does not account for changes in the composition or number of users in the event region (some studied events were quite remote and typically contained very few users).

Control 2 Another possibility is to simply follow the activity of the *same* users G_i from the event's cascade during normal time periods. This choice is keeps the population unchanged but it does not account for changes in who is being called; G_0 users may have chosen to call very different people during an emergency. Further, it does not account for the selection bias that is present during the event but not during the normal periods, which may exaggerate the change in call activity.

Control 3 Finally, one can study new cascades generated by the *same* event users G_0 during normal periods, creating a different cascade $\{G_0, g_1, g_2, \ldots\}$ for **each** normal period. The activities of each G_i can then be compared to those of the corresponding g_i's. This directly tests the effect that the initiating population G_0 has on the cascade, by studying those cascades the population would normally induce, and accounts for selection bias since this bias is present during the event and the normal periods.

We have chosen to use Control 3. Note that G_i will typically be larger than the normal g_i's and that G_i users may be more active than those in g_i, so $V(t|g_i)$ must be **rescaled** when being compared to the event's $V(t|G_i)$. To do this, we multiply $V(t|g_i)$ by a constant scaling factor a_i,

$$a_i = \int_{\delta t} V(\tau|G_i)\, d\tau \bigg/ \int_{\delta t} V(\tau|g_i)\, d\tau \,, \tag{S4}$$

where both integrals run over the same "calibration interval" δt and $\tau = 0$ is the start of the selection window. For most events we integrate over a 24-hour period two days before the window, $\delta t = (-48, -24)$. If the event is on a weekday, we ensure the calibration interval is not a weekend and vice versa. This factor a_i was chosen such that the total number of calls during normal time periods for $V(t|G_i)$ is approximately equal to $a_i V(t|g_i)$, equalizing the smaller time series and removing bias due to $|G_i| \neq |g_i|$.

Control 3 and Eq. S4 allow one to compare disparate populations' call activities, but there are two subtle yet important details to consider when comparing event and normal contact networks:

1. The event contact network under Control 3 consists of users $\left\{G_0^{\mathrm{e}}, G_1^{\mathrm{e}}, G_2^{\mathrm{e}}, \ldots\right\}$ while the contact network for normal period n contains users $\left\{G_0^{\mathrm{e}}, g_1^n, g_2^n, \ldots\right\}$. As shown in Fig. 1C, many members of G_0^{e} will be silent during each period n. This means that the cascade during period n is **actually** generated by a smaller set of users $g_0^{\mathrm{e}_n} \subset G_0^{\mathrm{e}}$, defined as those users in G_0^{e} who also make calls during period n. The rescaling factors a_i account for this when comparing activity levels $V(t|G)$ but this may give an unfair advantage to the growth of the event contact network, as quantified by the relative cascade size R, when compared to normal periods. For example, an event may result in $R > 1$ simply because the event period's "seed" population is larger than that of the normal period. See Fig. HA.

   To control for this effect, we compute G_0^{e} for the event and use it to generate $g_0^{\mathrm{e}_n}$ for each period n. We then return to the original event period and track a new event cascade for each $g_0^{\mathrm{e}_n}$. In other words, we consider only the smaller cascade $\left\{g_0^{\mathrm{e}_n}, g_1^{\mathrm{e}_n}, \ldots\right\}$ formed by following only users who are normally active during that time of day. This means there are now different event cascades corresponding to each normal period. See Fig. HB.

2. There is a tendency for non-emergencies to occur later in the day than the emergency events. Most concerts are held at night, for example. Users are generally more active during this time of day; many users have plans with free nightly minutes. This factor is not an issue for activity levels $V(t)$ since these are always compared during the same time of day. However, this may give an additional advantage when e.g., comparing R for an event that occurs early in the morning to an event that occurs during peak call activity. The latter cascade occurs when users are generally more active, increasing the number of potential propagation vectors and possibly increasing the rate at which new members are added to the G_i's.

   A simple way to control for this is the following. First, compute the Global Activity Level (GAL), the total number of phone calls in the entire dataset during each event's time window. Then, pick the event that contains the lowest GAL and shorten all the time windows (decreasing t_{stop} only) for the other events such that they have the same minimum GAL. Finally, generate the contact network during these reduced windows only. Doing this guarantees that every event has the same number of possible communications to propagate along. This process was used for all computations of R.

With this machinery in hand, we can now successfully demonstrate that there are anomalously large cascades for many emergencies, especially the bombing. Not only is $R > 1$ (Figs. 5c and H) but the contact populations show anomalous increases in call activity, even after all controls are in place (Figs. 3A, 3B,

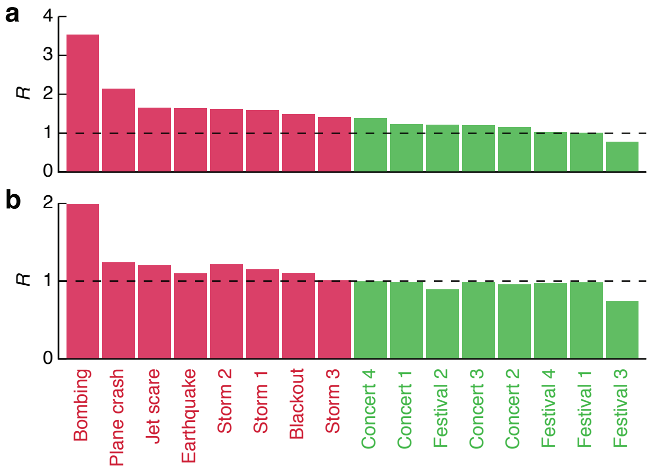

Figure H: **Controlling factors that affect the relative cascade size.** **A,** Relative cascade size $R = N_{\text{event}}/\langle N_{\text{normal}}\rangle$, where $N_{\text{event}} = \sum_i |G_i^e|$ and $\langle N_{\text{normal}}\rangle = |G_0^e| + \langle \sum_{i>0} |g_i^n| \rangle_n$ (averaged over normal events n). **B,** Relative cascade size using $R = \langle \sum_i |g_i^{e_n}| / \sum_j |g_j^n| \rangle_n$, controlling for the lower activity level during normal period n with $g_0^{e_n} \subset G_0^e$. With this control, all non-emergencies have $R < 1$ and all emergencies have $R \gtrsim 1$ (Storm 3 has $R = 1.0044$).

L, and M). All non-emergencies generate ordinarily-sized contact networks (Figs. 5c and H) and normal activity levels (Figs. 3A, 3B, N, and O).

		Event	GAL	t_{stop} (min)	t'_{stop} (min)
Emergencies	1	*Bombing*	2,327,592	120	65
	2	*Plane crash*	1,497,402	120	99
	3	*Earthquake*	1,370,268	60	60
	4	*Blackout*	4,536,253	180	51
	5	Jet scare	1,320,363	80	77
	6	Storm 1	3,701,872	135	48
	7	Storm 2	1,266,468	115	115
	8	Storm 3	2,654,849	120	51
Non-emergencies	9	*Concert 1*	8,136,294	360	46
	10	Concert 2	6,512,493	240	45
	11	Concert 3	10,384,830	540	49
	12	Concert 4	6,473,695	690	42
	13	*Festival 1*	13,782,768	960	74
	14	Festival 2	6,095,958	1,200	42
	15	Festival 3	19,504,204	1,200	90
	16	Festival 4	10,386,001	730	89

Table A: **Global Activity Level (GAL) and stop times for all sixteen events.** show the original t_{stop} and modified t'_{stop} that equalizes all events' GAL. Storm 2 had the smallest GAL over the corpus.

I Results on the event corpus

Sixteen events were identified for this work (see main text Table 1), but six events were focused upon in the main text. Here we report the results for all events. In Figs. I and J we provide the call activities $V(t)$ for all sixteen events used in this study (compare to Fig. 1). In Fig. K we show $\Delta V(r)$ for the ten events not shown in main text Fig. 2C. Finally in Figs. L, M, N, and O we present activity levels $V(t|G_i)$ for G_0 through G_3 for all 16 events.

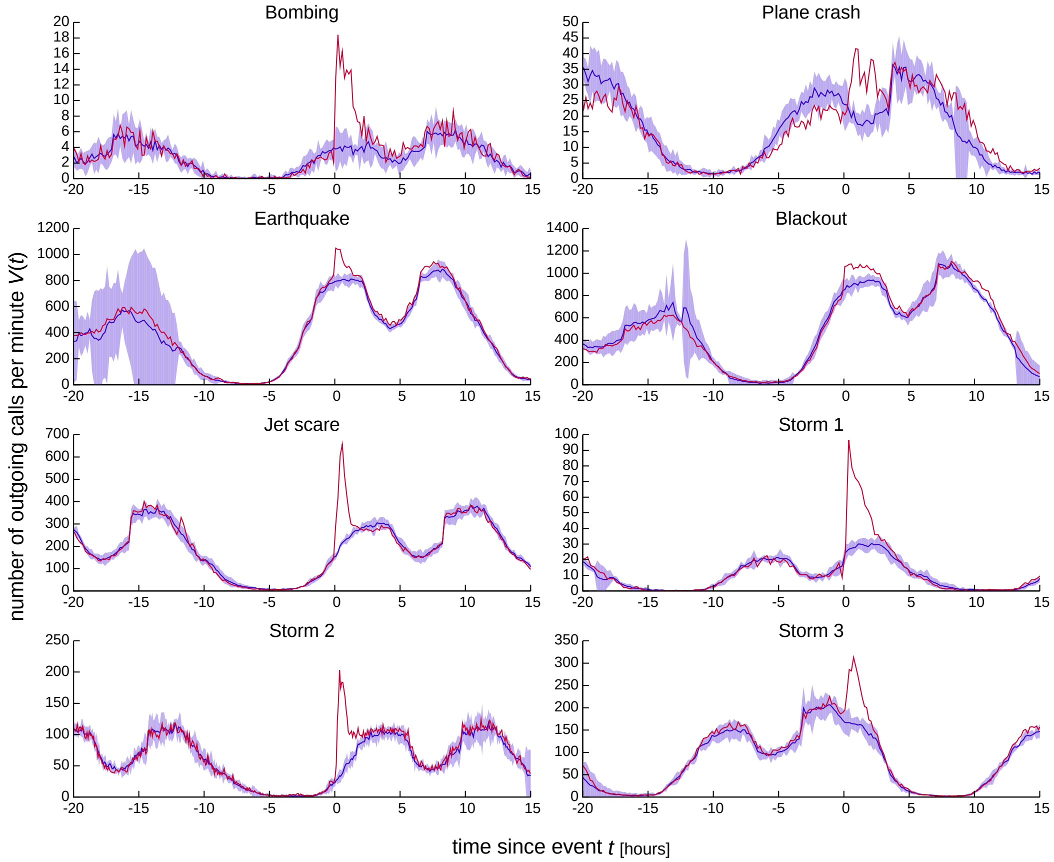

Figure I: Regional call activity for the eight emergencies analyzed. The first four are also shown in main text Fig. 1A. Shaded regions indicate ± 2 standard deviations.

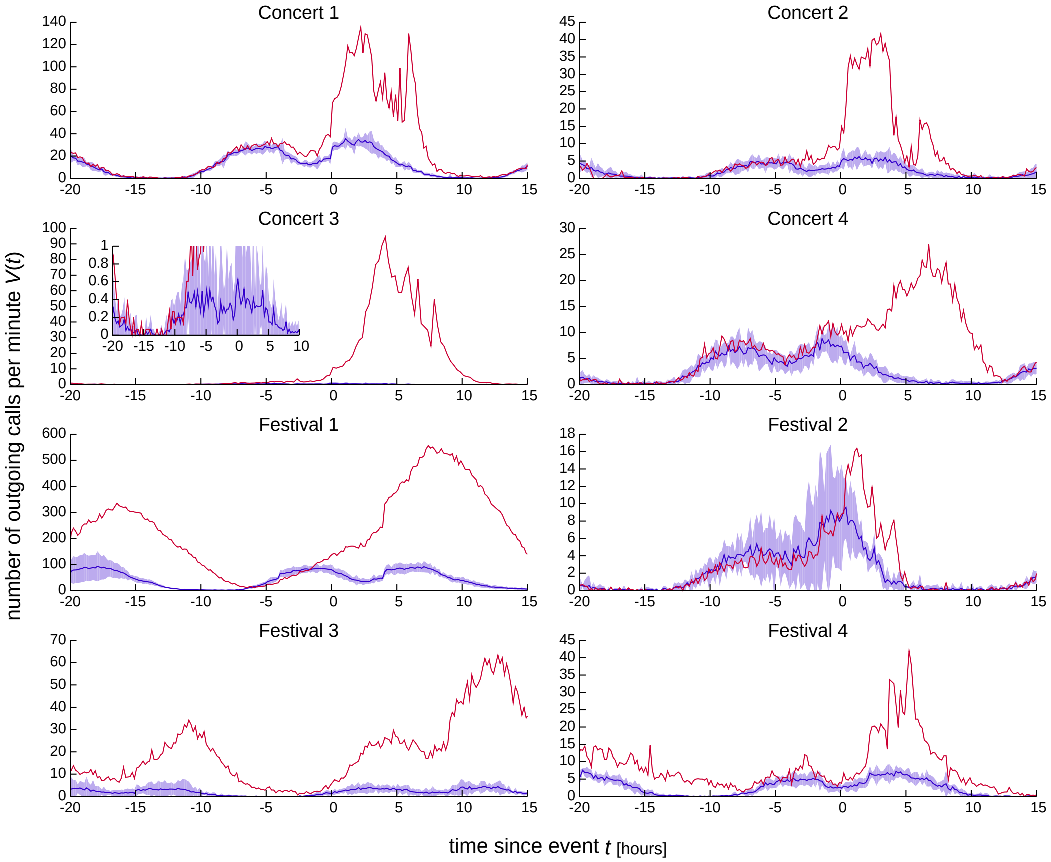

Figure J: The same as Fig. I for the eight non-emergencies. Concert 3 takes place at an otherwise unpopulated location and the normal activity is not visible on a scale showing the event activity.

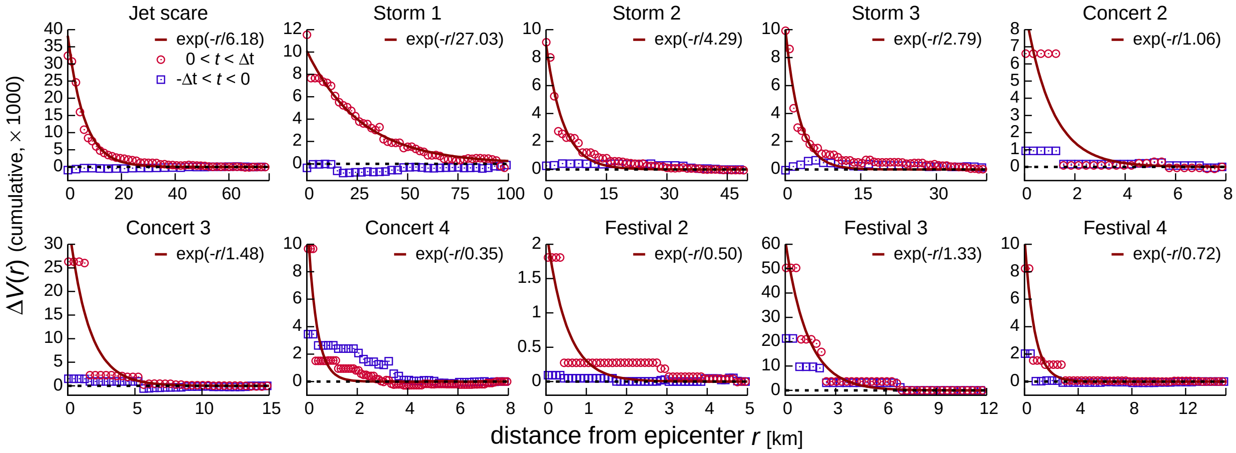

Figure K: Spatial call activity for the ten events not shown in main text Fig. 2C.

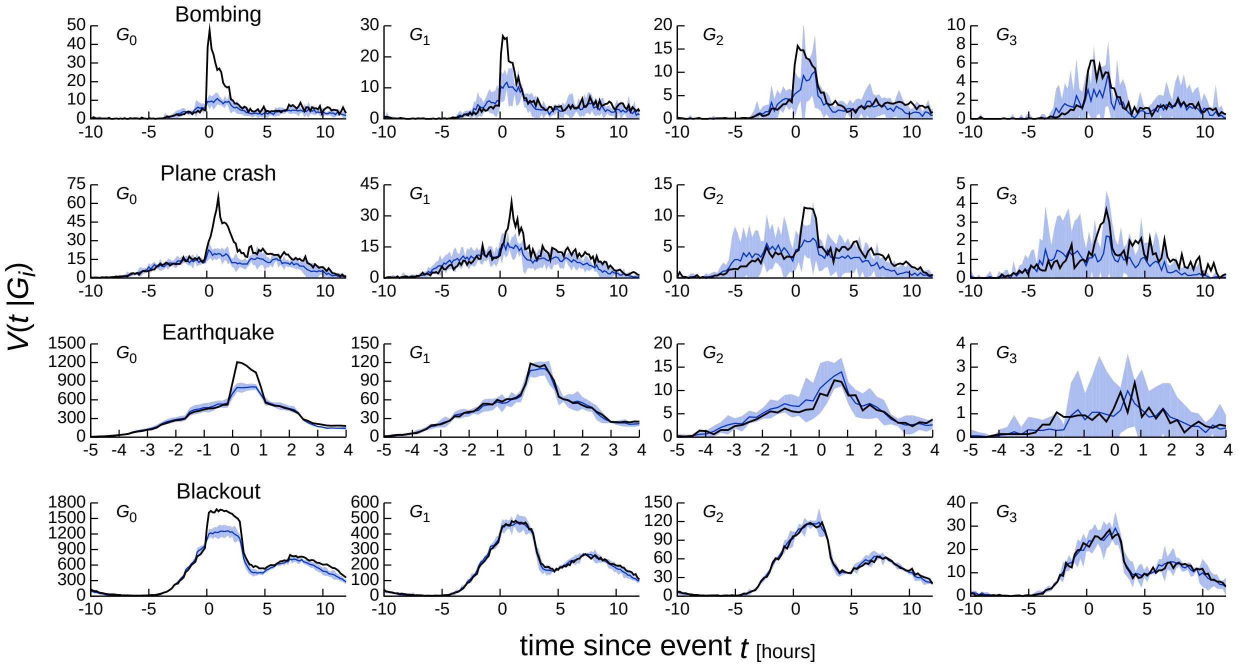

Figure L: Social propagation for the four main text emergencies. Shown are the activity patterns (conditional time series) for G_0 through G_3 during the event (black curve) and normally (shaded regions indicate ± 2 s.d.). Normal activity levels were rescaled to account for population and selection bias (see Sec. H) The bombing and plane crash show increased activities for multiple G_i while the earthquake and blackout do not.

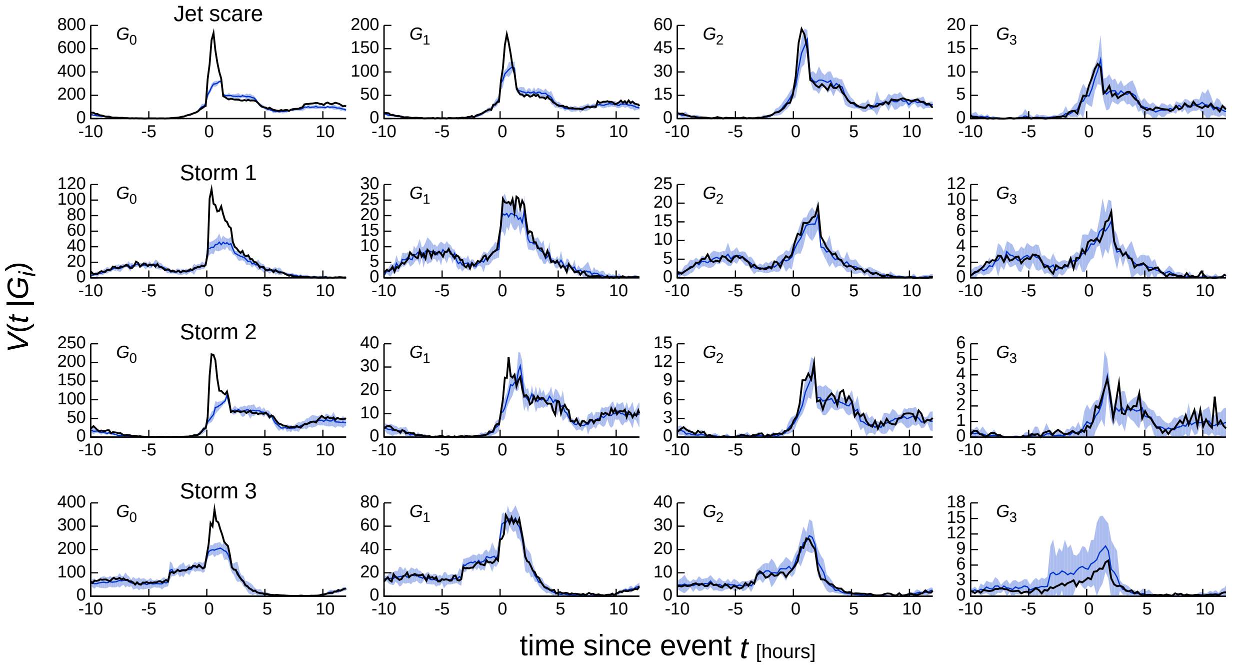

Figure M: Same as Fig. L for the remaining emergency events.

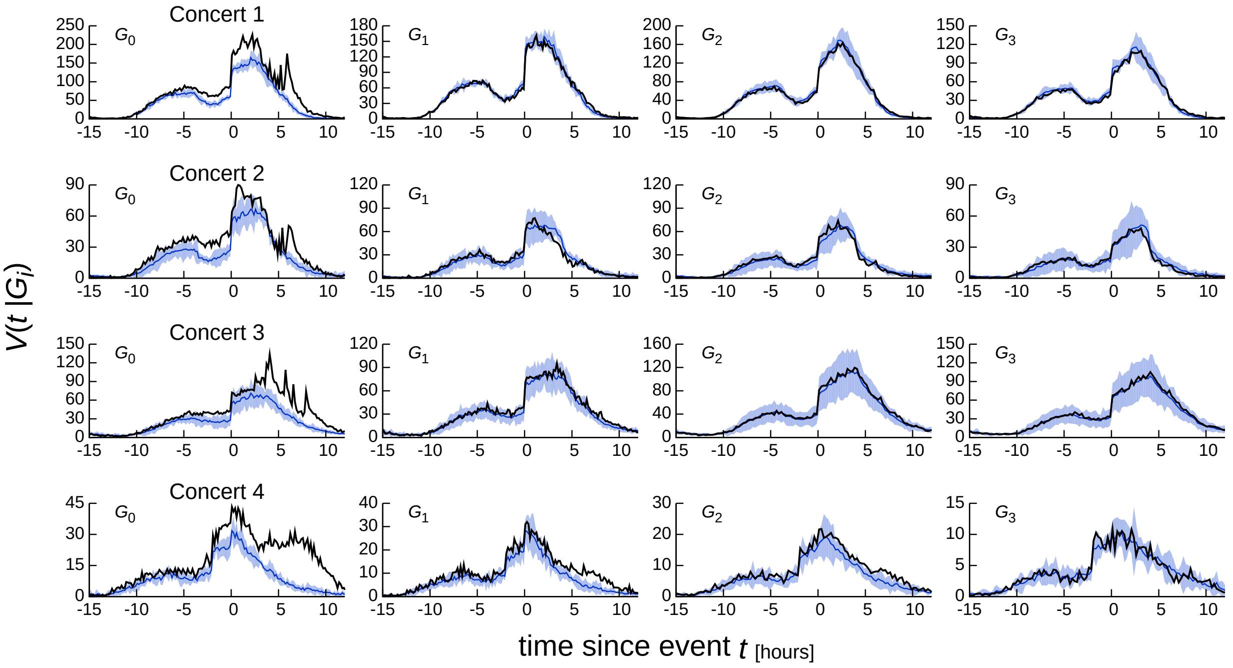

Figure N: Same as Fig. L for the concerts. All concerts show extra activity only for G_0 except Concert 4, which shows a small increase in activity for G_1 several hours after the concert started.

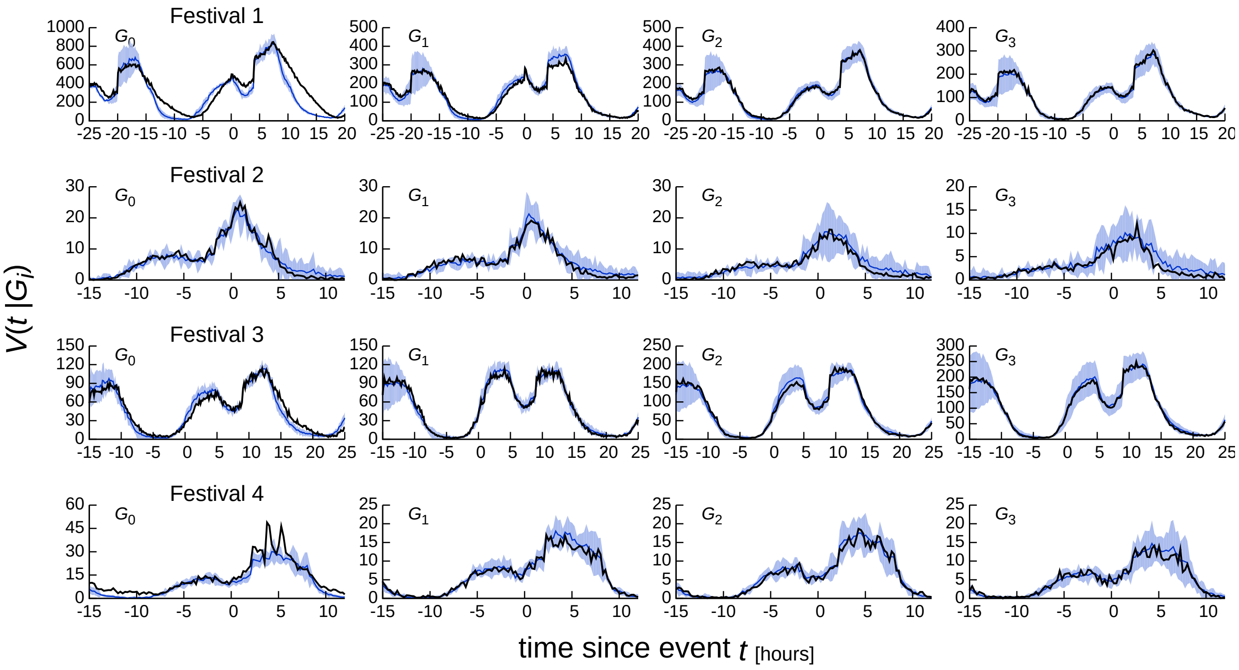

Figure O: Same as Fig. L for the festivals. Interestingly, Festival 2 shows no extra activity, even for G_0, indicating that the call anomaly for those events was caused only by a greater-than-expected number of users all making an expected number of calls.

References

[1] Onnela, J.-P. *et al.* Structure and tie strengths in mobile communication networks. *Proceedings of the National Academy of Sciences* **104**, 7332–7336 (2007).

[2] González, M. C., Hidalgo, C. A. & Barabási, A.-L. Understanding individual human mobility patterns. *Nature* **453**, 779–782 (2008).

[3] Song, C., Qu, Z., Blumm, N. & Barabási, A.-L. Limits of Predictability in Human Mobility. *Science* **327**, 1018–1021 (2010).

[4] Newman, M. E. J. The structure and function of complex networks. *SIAM Review* **45**, 167–256 (2003).
